# Glaucoma case finding and management among optometrists in Ghana and Nigeria

**DOI:** 10.1097/OPX.0000000000002283

**Published:** 2025-07-28

**Authors:** Obed Amoah-Smith, Justin Eghaghara, Barbara Ryan, David Grant Robinson

**Affiliations:** 1 Directorate of University Health Services, School of Optometry and Vision Science, University of Cape Coast, Cape Coast, Ghana; 2 Florida Eye Clinic, Wuse Abuja, Nigeria; 3 School of Optometry and Vision Sciences, Cardiff University, Cardiff, Wales, United Kingdom

## Abstract

**SIGNIFICANCE:**

This research contributes to the understanding of glaucoma case finding and management in Ghana and Nigeria. As areas of high glaucoma prevalence, gaining insight into this is paramount to identify barriers and better understand how to improve the detection of this blinding eye disease in an at-risk population.

**PURPOSE:**

This study was designed to investigate limiting factors to glaucoma case finding and management that exist within Ghana and Nigeria, including equipment and resource availability, management and referral options, optometrist skillset, and perceived socioeconomic barriers.

**METHODS:**

A descriptive, cross-sectional, online survey of licensed optometrists practicing in Ghana and Nigeria was conducted. The survey was distributed via an e-link and was designed to be self-administered. The survey consisted of 22 questions divided into three sections exploring (1) sociodemographic characteristics, (2) resources and skillset, and (3) case finding.

**RESULTS:**

A total of 318 optometrists (105 Ghanaians, 213 Nigerians) responded to the survey. Nearly all optometrists (>95% from each country) performed glaucoma case finding, diagnosed glaucoma, and prescribed antiglaucoma medications. The cost of performing clinical tests and antiglaucoma medications were readily cited as barriers to glaucoma care. Equipment availability for key clinical tests (Ghana %; Nigeria %, respectively) included slit lamp biomicroscope (94%; 76%), binocular indirect ophthalmoscopy lens (46%; 38%), Goldmann applanation tonometer (79%; 30%), and automated visual field analyzer (38%; 60%). At least half of the optometrists from both countries perceived the need for extra training on at least one glaucoma case finding technique.

**CONCLUSIONS:**

Optometrists from Ghana and Nigeria are involved in glaucoma case finding, diagnosis, and management. The main perceived barriers to optometric glaucoma care relate to the affordability of assessment/treatment and the need for additional training for some techniques. Equipment availability for key glaucoma investigations was also variable. Identifying and improving awareness of these barriers will facilitate strategies to improve the early detection and management of glaucoma in any nation that aims to promote health care system development.

Glaucoma is a multifactorial optic neuropathy that manifests through the progressive loss of retinal ganglion cell axons, structural changes of the optic nerve head, and progressive loss of vision.^1^ The exact pathophysiology of glaucoma is unknown. However, raised intraocular pressure (IOP) and/or vascular perfusion are implicated.^1^ Glaucoma is the second leading cause of blindness globally and the leading cause of irreversible blindness worldwide.^2^ It is projected that over the next 20 years, the number of people diagnosed with glaucoma worldwide will rise from ~80 million to ~110 million.^3^

It has been reported that there is more undetected glaucoma in Africa than in Europe, North America, Latin America and the Caribbean, and Oceania.^4,5^ Africa also has the highest documented prevalence of glaucoma (~5% of the population) globally, and in sub-Saharan Africa, the disease affects at least one in every 25 people aged 40 years and above.^5,6^ Within this region, the age of onset is lower and severity upon presentation is more advanced.^6,7^ The risk of progression to visual impairment can be mitigated via early detection and management. Despite the vision loss it causes, over half of those with glaucoma in sub-Saharan Africa are unaware. This is reflected in their late presentation, with 50% having already lost vision in one eye and at high risk of blindness by the time they seek treatment.^6^ As the vision loss caused by glaucoma is irreversible at this stage, there is nothing that can be done.

The predisposition of Africans to glaucoma and the high prevalence of advanced disease have been linked to multiple genetic, economical, and environmental factors.^5,6^ These include phenotypic characteristics, access to and provision of eye care, and the financial implications of long-term treatment. Glaucoma prevalence is particularly high in Ghana (6 to 7%) and Nigeria (5%), an area of combined 264 million population.^8–10^ Eye care services in these countries are still evolving. In both, the provision of eye care typically starts with a team of optometrists and/or ophthalmic nurses at a district level. Provision may be escalated to a regional level as required to engage the care of ophthalmologists, optometrists, and ophthalmic nurses who largely provide surgical services, clinical refraction, and outpatient services, respectively.

The minimum qualification required to practice as a licensed optometrist in Ghana and Nigeria is a Doctor of Optometry (OD) degree. This equates to approximately 205 semester hours of study. In addition to maintain an annual practice license, optometrists are also required to register with an optometry regulatory agency and the professional association, as well as meet continuing professional education requirements. The OD programs in Ghana and Nigeria are well established and became available in 2002 and 1980, respectively.^11^ Presently, two universities in Ghana and seven in Nigeria offer an OD program. The curriculum is largely harmonized across institutions and was founded on the four categories of clinical care model: optical technology services, visual function services, ocular diagnostic services, and ocular therapeutic services.^11–13^

Both didactic and practical methods are embedded into the curriculum. Teaching sessions cover many facets of the profession, such as clinical optometry, ocular disease, diagnostic imaging, pediatric optometry, low vision, public health, ocular pharmacology, and vision science. During their studies, students have practical tuition in the use of core instrumentation for glaucoma case finding. Equipment availability varies on an institutional basis. As a minimum standard, all students are taught slit lamp biomicroscopy, direct ophthalmoscopy, and applanation tonometry. The number of hours dedicated to refining each technique is also variable and is dependent on many factors, including infrastructure and resourcing.^11–13^ Presently, ultrasound pachymeters and gonioscopes are not readily available. Conversely, the accessibility of optical coherence tomography (OCT) and automated visual field assessment has improved over the past decade.

Academics in Ghanaian and Nigerian optometry institutions are predominantly optometrists with OD and PhD qualifications.^11,12^ Typically post-graduate qualifications held by these academics are research oriented. To boost staff strength, some universities retain their top graduates and attempt to mobilize support in terms of scholarships for them to pursue higher academic degrees. Currently, very few hold advanced qualifications in clinical subspecialties such as glaucoma. In some institutions, ophthalmologists also contribute to the delivery of teaching and assessment.

After optometry school, graduates complete a clinical internship within a hospital under the guidance of an experienced optometrist or ophthalmologist. This 1-year hands-on training period focuses on the diagnosis and management of diverse eye conditions. It is designed to not only enhance clinical skills but also cultivate confidence in delivering primary eye care services. Access to equipment to further enhance clinical skills during this period is variable.

Following completion of the internship, graduate optometrists in Ghana and Nigeria need to sit a professional licensure exam. Successful completion empowers them to provide general eye care services as well as diagnose and treat ocular disease, including glaucoma.^11–13^ Presently, post-graduate opportunities designed to further facilitate advanced studies and research also exist within Ghana and Nigeria. These include Master of Optometry (MOptom) and Master of Philosophy in Clinical Optometry or Vision Science (PhD) programs.

At the time of study, information from the respective Optometric associations revealed that there were approximately 500 and 5000 licensed and practicing optometrists in Ghana and Nigeria, respectively. Comparatively, there were far fewer ophthalmologists (91 and 700, respectively).^14,15^ Therefore, ensuring optometrists can case find and manage some glaucoma cases is essential.

A recent study profiling the glaucoma diagnostic practice of 493 optometrists from Ghana and Nigeria reported that a high proportion of respondents (>90%) diagnose glaucoma.^16^ The study highlighted that although these were the only African countries with the highest level of optometric education in Africa (World Council of Optometry Competency Level 4), the adoption of preferred practice guidelines and testing paradigms used was not consistent. The rationale for the variable adoption of guidelines such as those advocated by the International Council of Ophthalmology, American Academy of Ophthalmology, American Optometric Association, and the National Institute for Clinical Excellence remains unclear.^17–20^

The negative effects of visual impairment on the socioeconomic development of low- and middle-income countries are well documented. Early diagnosis and management of glaucoma will therefore be a positive step forward for any country that needs to promote development. This study has therefore been designed to further investigate limiting factors to glaucoma case finding and management that exist within Ghana and Nigeria, including equipment and resource availability, management and referral options, optometrist skillset, and perceived socioeconomic barriers.

## METHODS

This study was a descriptive cross-sectional survey of licensed optometrists practicing in private and/or public service in Ghana and Nigeria. These countries were targeted because both award a comparable Doctor of Optometry degree that enables a high degree of practitioner autonomy when assessing and managing glaucoma. This research was reviewed by three independent ethical review boards (Cardiff University School of Optometry and Vision Sciences Research Ethics Committee, reference 1599/1600; Institutional Review Board, University of Cape Coast, Ghana, reference UCCIRB/EXT/2023/21; Federal Capital Territory Health Research Ethics Committee, Nigeria, reference FHREC/2023/01/40/31-03-23).

The survey was developed using an electronic platform (Online Surveys; JISC 2021), which enabled digital design and distribution to respondents via an e-link. The link was disseminated to all members of the Nigerian Optometric Association and the Ghana Optometric Association via the distribution list of e-mail addresses held by each association. To drive engagement, the survey was also promoted at two continuing professional development events hosted at the University of Cape Coast in July 2023. An e-link was also posted onto established professional communication channels in both countries, including social media and digital messaging platforms. To mitigate repeat responses, participants were informed via a preamble not to complete the survey more than once or via multiple channels.

The questionnaire was designed to explore barriers to case finding (as opposed to detection). Both case finding and detection are strategies commonly used in healthcare and public health to identify individuals who may have a particular disease or condition, especially those who are not yet aware of their condition. Albeit these terms are often used interchangeably, case finding refers to a proactive approach that involves actively searching for potential cases. Conversely, detection refers just to the process of identifying and classifying a case. In the present context, we have chosen to use the definition of case finding as the study explores barriers to proactive assessment, diagnosis, and treatment strategy initiation for glaucoma in Ghana and Nigeria (as opposed to just opportunistic detection during routine eye examination). Also, as glaucoma in its early stages is asymptomatic, case finding is more appropriate for this study, as optometrists are required to adopt a strategy to actively search for individuals who have the condition, even if it is not their primary reason for attending.

The questionnaire (Appendix 1, available at https://links.lww.com/OPX/A842) was designed to be self-administered and anonymous, with an average completion time of 12 minutes as established via pilot study. It consisted mainly of multiple-choice questions and was based on verified tools from similar studies adapted for the target audience.^21,22^ Before progressing to the main body, participants were required to read a preamble. This provided an overview of the research, a link to a detailed participant information sheet, and a statement of informed consent. The main body had 22 questions divided across three sections. The first section collected sociodemographic characteristics relating to the participants’ education, mode of practice, location, and work environment. The second section focused on resources and skillset. Questions explored ophthalmic equipment used, drug availability, and self-reported confidence levels when performing key glaucoma diagnostic procedures. The final section elicited information relating to glaucoma case finding. This included questions exploring glaucoma diagnosis, prevalence, management strategies, and perceived barriers to the provision of care.

Using the formula for calculating sample size for cross-sectional studies/surveys,^23^ the minimum sample size for Ghana and Nigeria was calculated. A proportion of 50% of the population was assumed in this study (as no previous study had been published at the time of this study). Using the sample size formula,^23^ a precision of 5% with a 95% confidence level was used at a statistical significance level of 5%. The minimum sample size required for Ghana was 217 and that of Nigeria was 284. Data were initially stored on the platform's secure server before being exported into the statistical package for social sciences (IBM SPSS Statistics for Windows version 26, Armonk, NY) for analysis. Descriptive and inferential statistics (chi-square test of independence, p<0.05) were used to analyze the dataset in accordance with the primary aim.

## RESULTS

The online survey was launched in April 2023 and closed in September 2023. During this period, a total of 318 practicing optometrists in Ghana (n = 105, 33%) and Nigeria (n = 213, 67%) responded. Demographics of the respondents are summarized in Table 1.

**TABLE 1.** Characteristics of participating optometrists

	Ghana	Nigeria
	n (%)	n (%)
Years of practice		
1–5	35 (33%)	53 (25%)
6–10	31 (30%)	80 (37%)
11–15	38 (36%)	40 (19%)
>15	1 (1%)	40 (19%)
Mode of practice		
Public practice	57 (54%)	43 (20%)
Private practice	46 (44%)	161 (76%)
Locum	0 (0%)	4 (2%)
Not specified	2 (2%)	5 (2%)
Location of facility		
State capital	36 (34%)	127 (60%)
Other urban	54 (51%)	67 (31%)
Rural	13 (12%)	13 (6%)
Not specified	2 (2%)	6 (3%)
Post-graduate training in glaucoma		
Yes	7 (7%)	25 (12%)
No	94 (90%)	172 (81%)
Not specified	4 (3%)	16 (7%)

The ratio of respondents who had ≤10 years working experience compared with those who had been working as an optometrist for over a decade was 3:1. The minimum number of years qualified was 2 for each country and the maximum was 19 and 39 years for Ghana and Nigeria, respectively. Comparatively, the majority of respondents from Ghana (n = 58%) worked in the public sector, whereas a greater proportion from Nigeria were based in the private sector (n = 161, 76%). The proportion of optometrists who reported post-graduate training specific to glaucoma was low (7 and 12% of respondents from Ghana and Nigeria, respectively).

Respondents from Ghana and Nigeria reported an average number of patients examined per week as 174 and 89, respectively. A high proportion (>95%) of optometrists from both countries performed glaucoma case finding, diagnosed glaucoma, and prescribed antiglaucoma medications. It was estimated that around one in four patients seen had glaucoma or was a glaucoma suspect, with a slight preponderance for glaucoma to be more readily encountered by optometrists in Ghana than Nigeria (44 patients per month with glaucoma or glaucoma suspect in Ghana compared with 28 in Nigeria).

### Equipment and resource availability

Equipment availability for glaucoma case finding is shown in Table 2. Readily available equipment across both nations (Ghana %; Nigeria %, respectively) included visual acuity chart (100%; 99%), direct ophthalmoscope (96%; 93%), slit lamp biomicroscope (94%; 76%), mydriatic drugs (89%; 93%), and anesthetic drugs (89%; 90%).

**TABLE 2.** Availability of ophthalmic equipment and drugs for glaucoma case finding

	Ghana	Nigeria
	n (%)	n (%)
Intraocular pressure measurement		
Goldmann applanation tonometer	83 (79%)	64 (30%)
Noncontact tonometer	19 (18%)	136 (64%)
Perkins applanation tonometer	12 (11%)	68 (32%)
iCare tonometer	20 (19%)	45 (21%)
Schiotz tonometer	15 (14%)	53 (25%)
Anterior/posterior eye visualization		
Direct ophthalmoscope	96 (91%)	198 (93%)
Slit lamp biomicroscope	99 (94%)	162 (76%)
Binocular indirect ophthalmoscopy lens	48 (46%)	81 (38%)
Gonioscope	16 (15%)	49 (23%)
Headset binocular indirect ophthalmoscope	25 (24%)	51 (24%)
Fundus camera	13 (12%)	61 (29%)
Optical coherence tomography	43 (41%)	60 (28%)
Other instruments/resources		
Visual acuity chart	105 (100%)	211 (99%)
Automated visual field analyzer	40 (38%)	127 (60%)
Pachymeter	20 (19%)	86 (40%)
Mydriatic drugs	93 (89%)	198 (93%)
Anesthetic drugs	93 (89%)	192 (90%)

Clinical tests typically performed when investigating for glaucoma correlated with the availability of equipment in each country (Table 3). The most widely available method of measuring intraocular pressure in Nigeria was noncontact tonometry (68% optometrists performing this technique themselves). Comparatively, optometrist use of the Goldmann applanation tonometer was far more prevalent in Ghana (79%) than in Nigeria (30%). Equipment for measuring IOP (Ghana; Nigeria, respectively) also included Perkins (12%; 32%), iCare (19%; 21%), and Schiotz (14%; 25%) tonometers.

**TABLE 3.** How glaucoma diagnostic tests are obtained when indicated in Ghana and Nigeria

	Performed by me in my practice (n)		Performed by a colleague in the same practice (n)		No access to in practice, so not performed (n)		Patient referred elsewhere for the test (n)	
	Ghana	Nigeria	Ghana	Nigeria	Ghana	Nigeria	Ghana	Nigeria
GAT	75 (71%)	114 (54%)	8 (8%)	24 (11%)	8 (8%)	27 (13%)	14 (13%)	48 (23%)
Noncontact tonometry	18 (17%)	145 (68%)	1 (1%)	17 (8%)	47 (45%)	18 (9%)	39 (37%)	33 (16%)
Direct ophthalmoscopy	89 (85%)	198 (93%)	7 (7%)	8 (4%)	8 (8%)	2 (1%)	1 (1%)	5 (2%)
Slit lamp biomicroscopy	95 (91%)	139 (65%)	3 (3%)	28 (13%)	1 (1%)	18 (9%)	6 (6%)	28 (13%)
Volk lens BIO	33 (31%)	63 (30%)	15 (14%)	34 (16%)	38 (36%)	42 (20%)	19 (18%)	74 (35%)
Headset BIO	8 (8%)	23 (11%)	17 (16%)	38 (18%)	60 (57%)	63 (30%)	20 (19%)	89 (42%)
Gonioscopy	7 (7%)	27 (13%)	9 (9%)	44 (21%)	44 (42%)	67 (32%)	45 (43%)	75 (35%)
Fundus camera	8 (8%)	56 (27%)	5 (5%)	21 (10%)	45 (43%)	78 (37%)	47 (45%)	58 (27%)
OCT	28 (27%)	44 (21%)	15 (14%)	22 (10%)	7 (7%)	112 (53%)	55 (52%)	35 (16%)
Automated VFA	29 (28%)	119 (56%)	11 (10.5)	16 (8%)	17 (16%)	55 (26%)	48 (46%)	23 (11%)
Pachymetry	14 (13%)	77 (36%)	6 (6%)	16 (8%)	30 (29%)	75 (35%)	55 (52%)	45 (21%)

BIO = binocular indirect ophthalmoscopy; GAT = Goldmann applanation tonometry; OCT = optical coherence tomography; VFA = visual field analysis.

Less than half of the respondents from each country had access to a binocular indirect ophthalmoscopy (BIO) (Volk) lens (46%; 38%, Ghana; Nigeria, respectively), resulting in the technique only being performed by approximately one in three optometrists. Gonioscopes and pachymeters were also not widely available or used across both countries. Visual field analyzers were more common in Nigeria (60%) than Ghana (38%). Conversely, Ghanaian optometrists had better access to OCT in their facilities (41%; 28%, Ghana; Nigeria, respectively). Formal analysis (chi-square test, p<0.05) suggested that optometrists based in the Ghanaian private sector were significantly more likely to have access to an OCT (52% private vs. 28% public sector) and a visual field analyzer (50% private vs. 24% public sector) than those based in other work environments. However, when indicated, approximately half of the Ghanaian optometrists reported that they would refer a patient to another facility for OCT and visual field test when there is none in their facility. No such difference was found relating to equipment access for Nigerian optometrists based in the private and public sectors. Approximately 40 and 11% of Nigerians reported that they refer for OCT and visual field tests, respectively.

Respondents were asked to subjectively rate their confidence in performing and interpreting the results of key glaucoma tests (Table 4). Optometrists from both countries reported a high degree of confidence (>80% very confident) in performing direct ophthalmoscopy compared with other methods of fundoscopy. Approximately half of Ghanaian optometrists and a third of Nigerian optometrists reported very low levels of confidence performing BIO. More optometrists from Ghana (71%) were very confident in performing slit lamp biomicroscopy and OCT interpretation (50%) compared with Nigerian optometrists (32 and 35%, respectively). Conversely, optometrists from Ghana reported lower confidence levels in performing/interpreting headset BIO, fundus photography, automated perimetry, pachymetry, and gonioscopy compared with Nigerian counterparts.

**TABLE 4.** Confidence performing or interpreting key glaucoma tests in Ghana and Nigeria

	Not confident at all (n)		Not confident (n)		Just fine (n)		Confident (n)		Very confident (n)	
	Ghana	Nigeria	Ghana	Nigeria	Ghana	Nigeria	Ghana	Nigeria	Ghana	Nigeria
Applanation tonometry	5 (5%)	17 (8%)	4 (4%)	20 (9%)	16 (15)	32 (15%)	18 (17%)	41 (19%)	63 (59%)	103 (48%)
Direct ophthalmoscopy	3 (3%)	5 (2%)	0 (0%)	1 (1%)	1 (1)	5 (2%)	7 (7%)	21 (10%)	94 (90%)	181 (85%)
Slit lamp biomicroscopy	3 (3%)	11 (5%)	0 (0%)	22 (10%)	6 (6)	51 (24%)	21 (20%)	60 (28%)	75 (71%)	69 (32%)
Van Herick technique	25 (24%)	31 (15%)	0 (0%)	31 (15%)	14 (13)	39 (18%)	28 (27%)	41 (19%)	38 (36%)	71 (33%)
Smith’s technique	59 (56%)	52 (24%)	0 (0%)	34 (16%)	18 (17%)	50 (24%)	14 (13%)	35 (16%)	14 (13)	42 (20%)
Volk lens BIO	50 (48%)	68 (32%)	0 (0%)	36 (17%)	28 (27%)	44 (21%)	11 (11%)	28 (13%)	16 (15%)	37 (17%)
Headset BIO	70 (67%)	70 (33%)	0 (0%)	48 (23%)	16 (15%)	45 (21%)	9 (9%)	24 (11%)	10 (10%)	26 (12%)
Gonioscopy	74 (71%)	72 (34%)	0 (0%)	40 (18%)	17 (16%)	53 (25%)	10 (10%)	24 (11%)	4 (4%)	24 (11%)
Fundus photography	52 (50%)	42 (20%)	0 (0%)	35 (16%)	25 (24%)	34 (16%)	10 (10%)	31 (15%)	18 (17%)	71 (33%)
OCT interpretation	8 (8%)	13 (3%)	0 (0%)	21 (10%)	15 (14%)	47 (22%)	30 (29%)	57 (27%)	52 (50%)	75 (35%)
Visual field interpretation	16 (15%)	10 (5%)	0 (0%)	6 (3%)	9 (9%)	31 (15%)	29 (28%)	56 (26%)	51 (49%)	110 (52%)
Pachymetry	47 (44%)	26 (12%)	0 (0%)	20 (9%)	15 (14%)	29 (14%)	19 (18%)	31 (15%)	24 (23%)	107 (50%)

BIO = binocular indirect ophthalmoscopy; OCT = optical coherence tomography.

### Management and referral

The most widely used preferred practice pattern in use for glaucoma case finding and management across both countries was the American Optometric Association Guideline (Ghana 41%; Nigeria 30%). Outside of this, approximately 15 to 20% of optometrists followed either the International Council of Ophthalmology Guidelines or local hospital guidelines. About 30 and 15% of Ghanaian and Nigerian optometrists, respectively, reported using self-developed protocols. On average, each Ghanaian or Nigerian optometrist referred approximately 13 glaucoma-related cases per month. The most common management option (65%) by Ghanaian optometrists was to initiate treatment with no onward referral. Conversely, optometrists from Nigeria more readily initiated treatment while also referring to an ophthalmologist (51%). Referral options to other clinicians were similar across both countries, with ~20% referring patients to a partnering ophthalmologist practice, ~20% referring to a private ophthalmologist, ~20% referring to a government hospital, and ~10% referring to another optometrist skilled in glaucoma management.

The most routinely performed tests (Ghana %; Nigeria %, respectively) before commencing treatment or referral were assessment of optic disc using direct ophthalmoscopy (95%; 94%), OCT analysis of optic nerve head and/or retinal nerve fiber layer (90%; 61%), tonometry (applanation Ghana 77%; noncontact Nigeria 69%), and automated perimetry (60%; 74%). Pachymetry and Van Herick assessment of anterior chamber depth were most often routinely performed by Nigerian optometrists (pachymetry 55%; Van Herick 51%) compared with their Ghanaian counterparts (pachymetry 28%; Van Herick 45%). Volk BIO and gonioscopy were rarely performed by optometrists from both countries (Volk BIO Ghana 18%; Nigeria 21% and gonioscopy Ghana 7%; Nigeria 23%).

Only a small proportion of respondents (~5%) suggested that they would not repeat any tests before initiating treatment or referring for glaucoma. The most repeated tests (Ghana; Nigeria, respectively) were dilated assessment of the optic disc using direct ophthalmoscopy (80%; 78%), tonometry (applanation Ghana 71%; noncontact Nigeria 63%), and automated perimetry (51%: 57%).

Family history of glaucoma, optic nerve head profile, IOP measurement, and visual field profile were all commonly included (≥70%) in referral letters. Approximately 40% Ghanaian and 47% Nigerian optometrists included information on anterior chamber assessment.

### Barriers to glaucoma case finding

The main self-reported barriers to glaucoma case finding and management are related to the cost of assessment and treatment (Appendix Table A1, available at https://links.lww.com/OPX/A843). Optometrists from both countries reported that a patient’s ability to pay for the test or their willingness to pay for supplementary tests that may aid detection of glaucoma was a barrier (>60% agreed and strongly agreed). The cost of performing clinical tests and antiglaucoma medications was also readily cited as barriers to glaucoma care.

At least half of the optometrists from both countries perceived the need for extra training on some examination techniques, like Volk lens and/or interpretation of some test results, as a barrier. Less problematic were barriers relating to equipment availability, infrastructure, time constraints that may limit a clinician’s ability to conduct all necessary tests, and availability or access to training on glaucoma detection. A much larger proportion of optometrists from Ghana (99%) than Nigeria (50%) expressed an interest in attending training on glaucoma detection and management if it was available.

## DISCUSSION

This study sought to investigate barriers to glaucoma case finding and management within Ghana and Nigeria. The study has identified that involvement in glaucoma care, including diagnosis and medical management, in these nations was unequivocal. It is very clear that optometrists in Ghana and Nigeria have the equipment and skills to detect glaucoma. Most are able to manage glaucoma, but there are barriers to that. This is very encouraging given the large burden of glaucoma in these countries.

The primary self-reported barrier to glaucoma case finding and management is related to the cost of assessment and treatment, as opposed to equipment availability, optometrist skillset, and infrastructure. The perceived financial barriers reported impact all facets of glaucoma care, including baseline case finding, supplementary tests, and antiglaucoma medications. Respondents suggested that although the infrastructure was largely in place to facilitate glaucoma care and patients understood the importance, the delivery of optimum glaucoma care is limited by affordability.

The National Health Insurance Scheme (NHIS) in Ghana and Nigeria aims to reduce financial barriers to healthcare services. The scheme is well adopted in both countries and offsets some expenses relating to consultation fees and diagnostic tests. Presently, the only antiglaucoma medications for which the NHIS contributes toward cost are timolol eye drops and oral acetazolamide. Alternative topical hypotensives are available, but to gain access, patients are required to self-fund. The most widely performed glaucoma surgery is trabeculectomy, for which the NHIS typically funds a proportion of the cost. Conversely, newer interventions such as selective laser trabeculoplasty and minimally invasive glaucoma surgery are far less commonplace and not covered under the NHIS. Based on the outcomes of this study, the NHIS is not contributing enough to facilitate optimum glaucoma care in Ghana and Nigeria.

Access to equipment and resources within each country was in keeping with that reported by others.^16^ Albeit equipment availability was variable, both across and within the nations, access to instrumentation that enabled mainstay glaucoma investigations (ophthalmoscopy, tonometry, and visual field analysis) was widespread. The uptake of applanation tonometry was more prevalent in Ghana than Nigeria, where noncontact tonometry was more common; conversely, automated visual field analyzer availability was better in Nigeria (60%) than Ghana (40%). This availability is still well below United Kingdom (>95%), Ireland (87%), Australia (81%), and New Zealand (76%) counterparts.^24–26^

In both countries, even when equipment was available, confidence levels were often low and inhibitory with BIO, gonioscopy, and pachymetry. Despite widespread slit lamp availability, direct ophthalmoscopy appears to be the dominant method of assessing the optic nerve head. In alignment with this, it is reasonable to deduce that during glaucoma case finding in both countries, a patient is unlikely to undertake the gold standard triad of BIO, applanation tonometry, and automated visual field analysis as an achievable protocol. However, where accessibility issues existed or when confidence levels were low for a technique, referral pathways were widely utilized to enable a test to be conducted within a different facility or by another clinician.

The study revealed that after a glaucoma diagnosis, most Ghanaian optometrists (66%) commence treatment with no onward referral, whereas about half of the Nigerian optometrists initiate treatment before referring to an ophthalmologist. The reason for this discrepancy is unclear, especially when considering optometrists in both countries have similar qualification routes via respective Doctor of Optometry programs. We speculate that this difference may relate to increased equipment availability for gold standard investigations or increased involvement in glaucoma case finding by virtue of more Ghanaian optometrists being placed within better-resourced governmental facilities compared with Nigerian counterparts (54% of Ghanaian respondents based in public practice vs. 20% of Nigerian respondents). Other compounding factors include ophthalmologist availability and potential discrepancies in the cost of treatment within each country.^14,15^

The use of preferred practice guidelines was variable across both countries, with a generally low uptake compared with how readily therapeutic management was initiated. This reflects the level of autonomy of the glaucoma practitioner within these nations, where onward referral to an ophthalmologist to confirm diagnosis is not mandatory. There was, however, a strong preponderance to repeat clinical tests before initiating treatment. These findings align with the study conducted by Ocansey et al.,^16^ who suggested a need for streamlined national guidelines to ensure optimum evidence-based glaucoma care. In the present case, the most widely adopted guideline was that of the American Optometric Association. It is unclear whether awareness of this is a byproduct of the similar curriculum of optometry training programs between these two neighboring countries.

Optometry is a developing profession within Ghana and Nigeria, with the average number of years qualified by respondents being understandably low. There was also widespread recognition across both countries (Nigeria, 50% agree; Ghana, 61% agree) that additional training was necessary for some examination techniques and/or interpretation of some test results. However, training on glaucoma detection was cited as not readily available. Post-graduate training in both countries would improve glaucoma detection at least. Should more funding be forthcoming, this provides an opportunity to embed practices and protocols that set the foundation for optimum patient care for future generations.

There is also an onus for universities to continually upgrade the level of undergraduate optometric education and training to emulate the higher global standards of the profession. Periodic revision of programs utilizing information as reported in this study is paramount. This is to ensure that the next generation of optometrists are given adequate access to equipment and training in contemporary skills that will enable them to work at the top of their license. Better standardization of resource availability and harmonization of practical training during the OD program and internship would also mitigate against variability of the learning environment on a case-by-case basis.

The procurement of additional equipment and clinic space to enhance practical training is difficult. Since almost all optometry institutions in Ghana and Nigeria are state owned, the needed financial support must be forthcoming from appropriate government ministries. To supplement this, the proactivity of universities to identify other revenue streams and support the development of their programs is vital. For instance, funds generated internally from on-campus professional clinics or awarded through research funding could be put towards the cost of purchasing new equipment.

### Strengths and limitations

Albeit questions relating to hospital access and the development of local ophthalmology services elicited a positive response, a large proportion of optometrists who responded were based in urbanized areas. This is authentic and in keeping with evidence suggesting over half of optometrists in sub-Saharan Africa are based in this environment.^27^ The infrastructure in rural communities is not likely to mirror this and will be underserved in comparison. The reported number of ophthalmologists in Ghana and Nigeria is also low proportional to the population. In Nigeria, approximately 700 ophthalmologists serve a population of 228 million (1 per 325,000 people).^15^ Comparatively in Ghana, approximately 91 ophthalmologists serve a population of 34 million (1 per 375,000 people).^14^ Therefore, it is prudent to exercise caution when making inferences relating to the outcome of the present study and its relevance to equitable access to eye care in all regions.

The barrier relating to the cost of glaucoma treatment was investigated. However, drug availability was not. Responses relating to initiating treatment suggested that a high proportion of optometrists in both Ghana and Nigeria adopt a strategy that readily involves starting a treatment (with or without referral to an ophthalmologist). This suggests that drug availability may not be a larger barrier for most compared with affordability. However, regional variations in drug accessibility will exist and should be considered. Similarly, the study also did not explore the specific medications that optometrists from both countries prescribe and the factors that influence their choice of drug or management. This could have provided more information on what informs the glaucoma management pattern among optometrists in Ghana and Nigeria. Further research can be undertaken to explore this.

By virtue of the methodology used, this study is susceptible to selection bias. It is possible that more optometrists with an interest in glaucoma care or with better access to facilities may have responded to the survey. This would result in an overestimation in equipment accessibility and confidence when performing glaucoma-centric investigations. The subjective nature of this study is also a consideration, as it is for all questionnaire data. To verify true practice patterns or access to equipment, a clinical practice evaluation, such as that outlined by Shah et al.,^28^ would be valuable but was not feasible. Albeit only a small proportion of the optometrists invited to take part responded to the survey (20 and 5%, Ghana and Nigeria, respectively), the minimum sample size was reached. This does not provide assurance that the results are translatable to the wider profession; however, consistent findings with another survey conducted to explore glaucoma diagnostic practice in this region support the integrity of the data.^16^ Objective data on patient care would add further credibility to this study; however, that data is not presently available and has been identified as a future area to explore.

Despite these limitations, this study contributes to a growing evidence base relating to glaucoma care for a region where the disease is highly prevalent. The study has identified primary barriers inhibiting optimum glaucoma care in Ghana and Nigeria, highlighting inhibiting factors relating to affordability of care, availability of equipment, and the need for further training. It is hoped that the findings will be used by both educational providers and governmental bodies to target limitations that exist in their developing eye care systems.

## CONCLUSIONS

To our knowledge, this is the first survey to elicit Nigerian and Ghanaian optometrists' self-perceptions of barriers to glaucoma care and confidence when performing key investigations. The results from this study corroborate that optometrists in these countries readily diagnose and manage glaucoma. The main barriers to glaucoma care are related to affordability issues. However, equipment availability for enhanced case finding continues to be variable, with an overall preference for traditional direct fundoscopy as opposed to binocular indirect alternatives. Optometrists from both countries have high confidence in performing the most basic procedures required for glaucoma care. Training and investment targeting BIO, gonioscopy, and pachymetry would better align current protocols to the recommended preferred practice patterns.

Chronic open-angle glaucoma is highly prevalent, has an earlier age of onset, and is more aggressive with poor prognosis to treatment in Africans.^8–10^ This study adds value as it can be used to inform decision-making relating to workforce development and equitable access to eye care in Ghana and Nigeria.
